# Needs for robust research on thermo-electrochemical systems

**DOI:** 10.1093/nsr/nwag120

**Published:** 2026-02-27

**Authors:** Jorge García-Cañadas

**Affiliations:** Department of Industrial Systems Engineering and Design, Universitat Jaume I, Spain

Thermo-electrochemical systems combine electrochemical and thermal processes. Their main applications comprise energy harvesting (conversion of heat into electricity), energy storage (thermally chargeable supercapacitors) and sensing (temperature and thermal phenomena). Currently, the most-studied thermo-electrochemical systems are (i) thermogalvanic cells (TGCs) [1,2], (ii) ionic thermoelectric supercapacitors (ITESCs) [3] and (iii) thermally regenerative electrochemical cycles (TRECs) [4,5].

**Figure 1.**
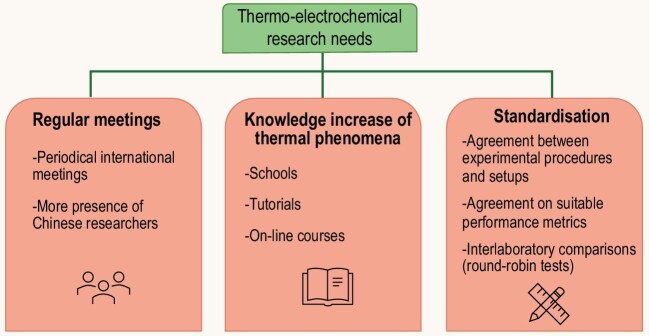
Summary of needs for robust research on thermo-electrochemical systems.

The thermogalvanic and Soret (thermodiffusion) effects occurring in TGCs and ITESCs, respectively, have been known for decades. However, TGCs and ITESCs have emerged in recent years following the publication of two influential studies [6,7]. On the other hand, decades ago, TRECs received significant attention, but focusing mostly on high temperatures (>500°C). It was in 2014 that a seminal article showed their application at near room temperature [8], which sparked renewed research interests. Despite the growth experienced in these fields, the research community working on thermo-electrochemical systems remains currently small, with the largest number of research groups located in Asia (mainly in China). In this perspective, I would like to highlight some of the needs (Fig. 1) that this small growing community requires in order to make thermo-electrochemical research strong, robust and respected.

First, it is important to meet, to establish regular meetings and conferences. Basically, all research areas have their own conferences. Research is multidisciplinary and this is especially true in thermo-electrochemical systems, which bring together disciplines such as inorganic/organic chemistry, electrochemistry, materials science, thermal engineering, polymer science and device engineering. Significant research advances occur when complementary expertise from different individuals converge, when knowledge is exchanged and when, by listening to others, one realizes that a topic previously considered irrelevant (or not considered at all) is in fact crucial. These things happen at conferences.

Efforts in this respect have started recently, with the International Workshop on Thermo-electrochemical Devices that celebrated its second

edition in December 2025. Its first edition, in September 2023, was the first international meeting exclusively devoted to the field of thermo-electrochemical systems. It brought together 30 participants (from Europe, Japan, South Korea and China) and the main topics were TGCs and ITESCs. In its second edition, 25 participants were present (from Europe, Japan, South Korea, China and the USA). Regarding the topics covered, again TGCs and ITESCs were dominant, with only one contribution about TRECs. A few days later, the Advances in Electrochemical and Liquid-based Thermal Technologies for Waste Heat Utilization and Electricity Storage symposium took place within Pacifichem 2025, with 14 contributions.

Apart from these international events, to my knowledge, only two national conferences took place in China, in May 2024 and May 2025, with an attendance of ∼120 participants in its second edition. This significant difference in the number of attendees (≤30 in the international workshop in Japan and >100 in the Chinese one) is worrying given the close proximity of both countries and it is important to have the Chinese community also present at international events. A possible reason for this is the political confrontation between both countries [9], which in fact was the reason for the cancelation of the participation of five Chinese researchers at the Japanese event. Other possible reasons could be the need for a visa to enter Japan or the difficulties of speaking English for some Chinese researchers. Apart from this, it is important to increase the presence of groups working on the TREC topic.

Another need I consider relevant is to strengthen the knowledge of the thermal part, as thermal phenomena are one of the main pillars in the field and knowledge of thermal engineering is often limited within research groups in this area, as most researchers come from disciplines such as chemistry, materials science, physics or chemical engineering, in which thermal transport is elusive or lightly covered. Understanding how thermocouples work, the best place to position them to accurately measure temperature, the temperature drops that might appear due to solid–solid interfaces, the need to use thermal interface materials, understanding of the heat transfer along the electrolyte (conduction and natural convection) and other thermal aspects are very important. Hence, I think it is key to try to improve this knowledge, which can be achieved through training by means of schools or tutorials (maybe the day before attending thermo-electrochemical systems workshops), online courses or books.

Standardization is another need, and quite urgent in my opinion. Each group uses its own setups and procedures and, given the significant number of variables that can influence the performance, different ways of measuring can lead to significantly different results. For example, in liquid TGCs, the orientation of the cell strongly affects the natural convection in the electrolyte. When the hot side is on top of the cold side, convection does not take place, but, in horizontal and cold-over-hot positions, it is significant and a higher power output is obtained. Also, the way in which the current–voltage curves are measured can significantly impact the power output. As the movement of ions is slow, it is important to give sufficient time for steady-state currents to be to reached and sometimes current values are not recorded under completely stationary conditions, which provides overestimated power outputs [10]. Another weak aspect is the way in which the efficiency of TGCs is provided, which estimates the heat input from the thermal conductivity of the electrolyte, instead of measuring the actual incident heat flux, which overestimates the efficiencies obtained [11].

It is very important to work on standardizing the ways in which performance metrics are achieved, otherwise the credibility of the field will be severely damaged and evaluation of the repeatability of the results becomes a very difficult task. Some steps that could be followed are: (i) to define a set of recommended cell geometries, (ii) to specify electrolyte orientation, (iii) to report current output vs time to ensure steady-state conditions, (iv) to define a suitable way to measure the heat power input and, ultimately, (v) to develop formal standards supported by interlaboratory comparisons and round-robin tests. A good example of efforts regarding standardization can be found in the photovoltaics area, where testing conditions are clearly established (AM1.5 illumination level, fixed cell areas, etc.) and external validators of results are in place, such as the National Laboratory of the Rockies, which provide certified efficiencies [12].

In conclusion, there are important needs that the thermo-electrochemical systems community should meet to make this research field strong and credible. Efforts on having meetings have started and continue, enhancing the knowledge of thermal phenomena is a key aspect to be covered and working on standardization is probably the strongest need to be addressed. Looking forward, considering the short term of a few years, the thermo-electrochemical community should make progress in achieving broader international participation (continue the current international workshops in suitable locations and maybe perform some meetings also online). Also, training courses or video tutorials addressed at PhD students and newcomers should be run. Moreover, progress can be achieved in reaching common evaluation metrics and setup designs. Finally, it is also relevant to set clear application-driven benchmarks, such as establishing efficiency thresholds, suitable charging times and cycle-stability metrics for applications such as the internet of things.
